# Underlying lung disease and exposure to terrestrial moderate and high altitude: personalised risk assessment

**DOI:** 10.1186/s12890-022-01979-z

**Published:** 2022-05-09

**Authors:** Kirsten Holthof, Pierre-Olivier Bridevaux, Isabelle Frésard

**Affiliations:** 1 Service de pneumologie, Centre Hospitalier du Valais Romand, Avenue du Grand-Champsec 80, 1950 Sion, Switzerland; 2 Service de pneumologie, Hôpitaux universitaires de Genève, 1211 Geneva 14, Switzerland; 3 Geneva Medical School, University of Geneva, Geneva, Switzerland

**Keywords:** Chronic lung disease, High altitude travel, Air travel, Exercise performance, Prediction tools, Hypoxic tests

## Abstract

Once reserved for the fittest, worldwide altitude travel has become increasingly accessible for ageing and less fit people. As a result, more and more individuals with varying degrees of respiratory conditions wish to travel to altitude destinations. Exposure to a hypobaric hypoxic environment at altitude challenges the human body and leads to a series of physiological adaptive mechanisms. These changes, as well as general altitude related risks have been well described in healthy individuals. However, limited data are available on the risks faced by patients with pre-existing lung disease. A comprehensive literature search was conducted. First, we aimed in this review to evaluate health risks of moderate and high terrestrial altitude travel by patients with pre-existing lung disease, including chronic obstructive pulmonary disease, sleep apnoea syndrome, asthma, bullous or cystic lung disease, pulmonary hypertension and interstitial lung disease. Second, we seek to summarise for each underlying lung disease, a personalized pre-travel assessment as well as measures to prevent, monitor and mitigate worsening of underlying respiratory disease during travel.

## Introduction

Rapid evolution in travel options by expanding road, rail, cable cars and airway networks have made moderate to high altitude destinations increasingly accessible and affordable for people of varying age, health condition and fitness. Older patients constitute a significant part of the population in mountain resorts located at moderate to high altitude (1500–2500 m). A tourist enquiry in 2015 showed that 11.8% of Valais visitors is over 65 years of age [1]. Although age in itself is not a risk factor for altitude intolerance, older patients are more prone to pulmonary comorbidities.

Previous literature has shown that all individuals travelling to moderate (> 1500 m) or high altitude (> 2500 m) regions are at risk of experiencing dyspnoea, disturbed sleep with periodic breathing and exercise limitation, especially when combined with rapid ascent. Acute mountain sickness (AMS), with headache, gastrointestinal symptoms, weakness and sleeping difficulties, in the mild form to acute high altitude cerebral oedema (HACE), in the most severe form, as well as high altitude pulmonary oedema (HAPE) can develop when travelling to high altitude [2].

Growing awareness of the possible health risks of an altitude sojourn motivates a rising number of people to seek medical advice prior to travel.

Consequently, clinicians are regularly confronted with difficult questions about the health risks of mountain travel or altitude sojourn by their patients. In the field of respiratory medicine and altitude, the central goal of personalised risk assessment is to yield individual estimates of adverse health outcomes, acknowledging the limitations of clinical evidence, the heterogeneity of chronic lung diseases and comorbidities, as well as the risks associated with moderate altitude. Personalised risk assessment also integrates patients’ values and preferences as well as shared decision making.

It may help patients with underlying chronic respiratory conditions to adopt health behaviors allowing safer travel to moderate altitude.

First, this review aims to summarise the effects of moderate to high terrestrial altitude (1500–4000 m) exposure on patients with pre-existing lung disease, more specifically chronic obstructive pulmonary disease (COPD), sleep apnoea, asthma, bullous or cystic lung disease, pulmonary hypertension and interstitial lung disease. Second, we seek to summarise for each underlying lung disease, a personalized pre-travel assessment using appropriate tests as well as measures to prevent, monitor and mitigate worsening of underlying respiratory disease during terrestrial altitude travel.

## Methods

We performed a comprehensive literature search using the databases Pubmed, Google Scholar, Web of Science, citations and references of retrieved articles, from 1970 to 2021 with the following keywords ((((((((((COPD)) OR (asthma)) OR (pulmonary hypertension)) OR (sleep apnoea syndrome)) OR (chronic lung disease)) OR (pneumothorax)) OR (cystic lung disease)) AND ((high altitude) OR (moderate altitude))). After exclusion of published reviews and screening of the references and citations of identified articles, 137 were considered for the present review. We found 13 randomized controlled trials, 33 observational studies and 3 case reports.

### Physiological effect of moderate to high altitude and hypoxia in patients with chronic respiratory conditions

The most significant change at moderate to high terrestrial altitude is the exponential drop in barometric pressure resulting in a lower partial pressure of inspired oxygen (PIO_2_), partial pressure of alveolar oxygen (PAO_2_) and arterial oxygen pressure (PaO_2_), also referred to hypobaric hypoxia [3–5]. Table 1 provides an overview of the altitude related barometric changes with estimated Pb, PAO_2_, PaO_2_, PaCO_2_ and SpO_2_ with increasing terrestrial altitude. To the best of our knowledge, no study specifically addressed the gas exchange adaptation after acclimatization at moderate altitude. Brothers et al. [6] examined the hematological and exercise adaptation to moderate altitude at 46 weeks in younger healthy subjects without measuring arterial blood gases (ABG). Oxygen content was studied by Grocott et al. on Everest climbers. A fall in PaO2 was demonstrated at high altitude compensated by hemoglobin increase and thus relatively preserved oxygen content. Unfortunately ABG analyses were not performed at moderate altitude [7].

**Table 1:** Overview of estimated *Pb, PAO_2,_ PaO_2_, PaCO_2_, SpO_2_ with increasing terrestrial altitude. Adapted from [4, 7, 8]

Altitude (m)	Examples	Pb (mmHg)	PAO_2_ (mmHg)	PaO_2_ (mmHg)	PaCO_2_ (mmHg)	SpO_2_ (%)
Sea level	–	760	149	90–100	38–42	97–99
1500	Verbier (CH)	630	122	65–80	33–42	93–97
2000	Saas-Fee (CH)	600	116	64–67	33–38	90–96
2500	Machu Pichu (PE)	560	108	45–70	31–36	88–95
3000	Lukla (NP)	525	100	49–54	31–35	84–92
3500	La Paz (BO)	490	93	42–53	32–34	80–89
8840	Everest summit (CN/NP)	253	43	27–32	10–14	54–62

*CH* Switzerland, *PE* Peru, *BO* Bolivia, *NP* Nepal, *CN* China, *Pb* barometric pressure, *PAO*_***2***_ partial pressure of alveolar oxygen, *PaO*_***2***_ partial pressure of arterial oxygen, *PaCO*_***2***_ partial pressure of arterial carbon dioxide, *SpO*_***2***_ arterial oxygen saturation

Acclimatization helps to maintain adequate tissue oxygen delivery at high altitude [2, 4]. Some of the physiological responses to hypobaric hypoxia occur almost immediately, whereas others need hours to even days to reach their full expression.

Among these adaptive responses, the need to increase ventilation, a mechanism known as the hypoxic ventilatory response (HVR) leads to a greater utilisation of an individual’s breathing reserve [9]. Normal gas exchange capacity is also a key element to maintain adequate oxygen delivery when exercise is performed at altitude. Thus, patients having chronic respiratory conditions with ventilatory or gas exchange limitations are obviously at risk of severe hypoxemia. In clinical practice, measurement of carbon monoxide transfer capacity (TLCO) is the most available method to evaluate potential gas exchange limitation.

A third phenomenon, hypoxic pulmonary arterial vasoconstriction (HPV) may be deleterious in patients with chronic respiratory conditions, who may have some degree of pulmonary hypertension at sea level, as it results in a substantial increase of the pulmonary vascular resistance and pulmonary arterial pressure (PAP), potentially causing right heart failure. HPV plays an important role in the development of HAPE in healthy subjects [4, 8].

**Fig. 1:**
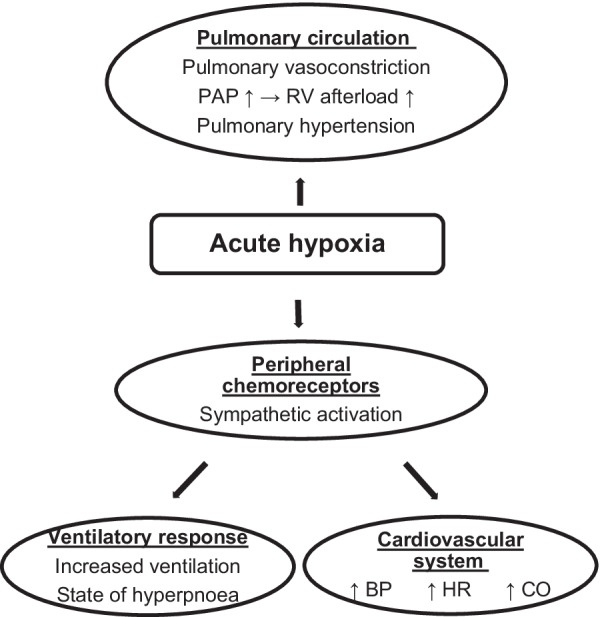
Physiological responses to altitude induced hypoxia in healthy individuals. Adapted from [10]. *PAP* pulmonary arterial pressure, *RV* right ventricle, *BP* blood pressure, *HR* heart rate, *CO* cardiac output

Figure 1 provides an overview of the physiological response mechanisms when a healthy individual is exposed to altitude induced hypoxia.

We hereafter review the effect of altitude exposure in six common respiratory conditions.

### Chronic obstructive pulmonary disease

COPD is the most frequent chronic respiratory disease, with a prevalence of 6.1% in men and 4.0% in women in Switzerland [11].

COPD is characterised by chronic airflow obstruction, reduced carbon monoxide transfer capacity, impaired pulmonary gas-exchange and ultimately hypoxemia. Exposure to a hypobaric hypoxic environment can cause an aggravation of gas exchange limitations requiring careful assessment in severe cases.

Limited data are available to predict the effect of hypoxia on the resting state as well as exercise performance of COPD patients at altitude. The existing literature demonstrates that patients with moderate to severe COPD experience worsening hypoxaemia and dyspnoea when exposed to a hypobaric hypoxic environment [12–19].

Two recent randomized crossover studies included moderate to severe COPD GOLD grade 2–3 patients (FEV1 30–80% predicted), living below 800 m, and exposing them sequentially to altitudes of 1650 m and 2590 m during a 4-day sojourn with supervised cardiopulmonary exercise test and 6 min walking distance (6MWD) [17, 18].

A significant reduction of 54% in submaximal exercise endurance time at 2590 m as compared to 490 m was found in one study. The decreased performance at 2590 m was associated with arterial hypoxaemia and deoxygenation of both the muscle and cerebral tissues (measured by near infrared spectroscopy), as well as an impaired cerebral blood flow response (measured by transcranial Doppler ultrasound).

The authors concluded that the major limitation at exercise was most likely due to a reduced systemic and cerebral oxygenation rather than ventilatory constraints. This suggests that O_2_ supplementation can be an effective measure when planning exercise during altitude travel [18].

In another crossover study, 6MWD was mildly impaired at moderate altitude (− 41 m) as well as maximal exercise performance (− 7%) [17].

Despite performing a physical activity up to exhaustion, none of the patients in the two studies experienced serious adverse events without O_2_ supplementation. The altitudes chosen for the studies were of clinical interest, as tourists frequently visit an altitude of 1500–2500 m during leisure activities such as hiking or skiing. It is also close to the maximum altitude equivalent of cabin pressure in commercial air travel [20].

### Pulmonary hypertension secondary to chronic lung disease or chronic hypoxaemia

Pulmonary hypertension is a frequent complication in patients with chronic parenchymal lung disease, due to tissue destruction with loss of pulmonary capillary bed. A sustained increase of the mean pulmonary artery pressure (PAP) might ultimately lead to right ventricular failure [13]. As mentioned earlier, altitude hypoxia triggers further hypoxic pulmonary vasoconstriction.

Cardiac function and pulmonary haemodynamic changes were assessed by echocardiographic imaging at 490 m and after one night at 2590 m in a cross-over randomized trial including moderately to severe (GOLD 2–3) COPD patients [13]. An increase in systolic PAP (28 mmHg at 490 m to 39 mmHg at 2590 m) and indications of cardiac functional changes, with increased systemic systolic pressure, minor reduction in left ventricular ejection fraction (LVEF), and signs of right ventricular (RV) dysfunction were found. However, short exposure to altitude was generally well tolerated in terms of symptoms. Whether the sustained increase in PAP and RV dysfunction in a more prolonged exposure to altitude without O_2_ supplementation can be tolerated, is not known.

### Asthma

Asthma is a common chronic respiratory disease, affecting about 7% of the adult population [11].

Previous literature reports a potential benefit of long-term stays at moderate altitude (> 1500 m) for asthmatic patients most likely due to the lower exposure to aero-allergens. The inverse association between asthma incidence and altitude aligns with the fact that symptom control, lung function and local airway inflammation (total Immunoglobulin E, FeNO and blood eosinophil count) improve significantly when patients move to a moderate altitude location. These improvements have been attributed to the lower burden of house dust mite allergens [21–24]. Environmental changes occurring with increasing altitude, with lower outdoor temperature and lower humidity levels prevent the development of house dust mites.

Nevertheless, asthmatic conditions improve irrespective of sensitisation status to pollen or house dust mites at moderate altitude suggesting that there are also other beneficial contributing factors. Indeed, ascent to higher altitude is associated with decreased air density and reduced respiratory resistance with increased inspiratory and expiratory flow rates [21, 25, 26]. The fact that asthmatic patients have better symptom control with prolonged stays at high altitude does not imply similar outcome for short stays. Short-term visitors with asthma are exposed to a complex interplay between multiple factors that influence asthma control: exercise, hypoxic environment, cold and dry air possibly leading to increased bronchial hyper-responsiveness (BHR) and exercise induced bronchoconstriction (EIB) [26, 27].

The direct effect of hypoxia per se on airway function and bronchial reactivity remains unclear. Mild hypoxia (FiO_2_ 15%, simulating 2438 m) induces BHR, while more profound hypoxia (FiO_2_ 10%, simulating altitude > 5800 m) stimulate the release of catecholamines and cortisol, both having a protective effect on BHR [26].

An important trigger for increasing BHR and asthmatic symptoms is cold air. Studies show a higher prevalence of asthmatic symptoms and EIB in cross country skiers and mountaineers, who spend a large amount of their time exercising in cold air [28, 29]. Cold air is probably the most important contributing factor to loss of asthma control at high altitude, due to damaged airway epithelium with increased neutrophilic airway inflammation and increased airway obstruction [27].

EIB is a distinct manifestation of BHR with transient narrowing of the airways and fall in FEV1 after exercise. EIB is more common in patients with asthma but can also occur in individuals without underlying asthma [30, 31]. The main pathophysiological mechanism is most likely mediated by airway dehydration, caused by increased ventilation during exercise and resulting in augmented osmolality of the airway lining fluid. This process triggers the release of inflammatory mediators, causing smooth muscle contraction and airway oedema. Cold air is another proposed mechanism, triggering an initial constriction of the bronchial micro vessels during exercise followed by hyperaemia when no longer exposed to cold air [32].

EIB is not per se due to the exercise itself, but rather due to the cooling and drying of the airways because of increased ventilation [33]. Travelling to altitude is thus an “exercise challenge test” in itself for the asthmatic patient. Hypoxic induced hyperventilation leads to increased ventilation of cool and dry air, irrespective of the level of exertion, possibly causing bronchoconstriction. Any additional exercise at altitude causes an even greater increase in ventilation enhancing the risk of EIB.

Uncontrolled baseline airway inflammation can exaggerate this response, increase underlying EIB and finally expose the asthmatic patient to an altitude-induced exacerbation [30].

### Asthma field studies

Because of the heterogeneity of asthma between patients and the within-patient intrinsic variability of the disease, it has been difficult to design studies including asthmatic patients at altitude. Allegra et al. measured BHR in eleven mild asthmatic patients during two mountaineering expeditions, one at 4559 m in Capanna Margherita (Monte Rosa, Italy) and another one at 5050 m near the Mount Everest base camp in Nepal. Their results show a reassuring reduction in BHR at high altitude [34].

In another report of 10 healthy controls and 5 mild well-controlled asthmatic patients participating in a 2-week trek in the Himalayas, a significant decrease in the peak expiratory flow (PEF) was seen in asthmatics at the two highest altitudes, 3500 m and 4100 m compared to the control group. In addition, a significant fall in post-exercise arterial oxygen saturation was seen in the asthmatic group, a change that was not seen in the non-asthmatic group. The report suggests that even mild well-controlled asthmatic patients are more prone to develop high altitude hypoxaemia, predominantly due to bronchoconstriction induced by increased ventilation of cold and dry air [33].

An epidemiological study found that pre-travel frequent use of rescue bronchodilators and participation in intense physical activity exposed travellers to a higher risk to develop an asthma attack at altitude [35].

Stokes et al. prospectively studied 18 asthmatic patients versus 291 non-asthmatic controls undertaking a summit attempt of Mount Kilimanjaro (5895 m). Their study showed no significant differences in physiological parameters (heart rate, respiratory rate, oxygen saturation and PEF) between asthmatic patients and controls on ascent. The incidence of AMS and summit success rate were similar in the two groups [36].

In summary, these field studies provide reassurance that well-controlled asthmatic patients can ascent safely to 5000 m without significant adverse effects. However, caution must be taken in generalising these results to patients with severe asthma, as only subjects with mild asthma were included in these field studies. Thus, broncho-provocation testing, by means of a ‘high intensity exercise’ test or ‘eucapnic voluntary hyperpnoea’ (EVH) test as well as a hypoxic challenge test could be advised in patients with severe asthma planning a expedition in high altitude destinations.

### Sleep apnoea syndrome

#### Obstructive sleep apnoea syndrome (OSAS)

OSAS, defined as repeated episodes of apnoea/hypopnoea during sleep, is a prevalent syndrome affecting 5–10% of the adult population [37]. Continuous positive airway pressure (CPAP) is currently the most effective treatment option to improve breathing disturbances, sleep quality and daytime symptoms [38]. An alternative treatment option can be a mandibular advancement device [39].

When travelling to altitude destinations, carrying along a CPAP device can be quite inconvenient. Practical issues such as power supply to keep the device charged may render patients with known OSAS unable to continue CPAP therapy during altitude sojourns.

#### Physiological consequence of altitude in OSAS

A crossover trial included 34 OSAS patients, left untreated, with median apnoea/hypopnoea index (AHI) of 47.5/h and living below 600 m, in a study at 490 m, at 1860 m and at 2590 m respectively. Full night polysomnography, symptom questionnaires (Lake Louise Score and Karolinska Sleepiness Score) and driving simulator tests for evaluation of vigilance and reaction time were performed.

Results show an increase of the mean AHI on the second night at moderate altitude (90.9 events/h at 2590 m). Higher AHI was driven by increased central apnoea/hypopnea events at higher altitude without change in the rate of obstructive events. This suggests that acclimatization does not affect the rate of obstructive apneic events in patients with OSAS. Sleep quality was impaired and daytime evaluation showed a decrease in psychomotor vigilance during driving simulator tests. Increasing altitude also induced cardiovascular stress with increased systolic blood pressure and cardiac arrhythmias [40].

These data suggest unfavourable acute health effects in OSAS patients if left untreated when travelling to moderate to high altitude, risking aggravating hypoxaemia, increased central sleep events, impaired daytime performance and increased cardiovascular stress.

### CPAP and alternative treatment options for OSAS at altitude

Acetazolamide has been evaluated as a possible alternative treatment option during altitude sojourns. Acetazolamide, a carbonic anhydrase inhibitor, improves high altitude periodic breathing and prevents and treats acute mountain sickness in healthy subjects [41, 42]. Acetazolamide (250 mg twice daily) significantly improved nocturnal oxygenation, with reduction of central sleep events, improved sleep quality and sleep efficiency when compared to no treatment at all. Acetazolamide also prevented excessive blood pressure rise and weight gain at altitude. Obstructive sleep events were however not affected and no significant effect on daytime performance was revealed [43].

If CPAP therapy cannot be used or is refused, acetazolamide can hence benefit OSAS patients travelling to attitude by reducing their central apnoea index.

Latshang et al. examined if the combined use of acetazolamide with auto-CPAP (auto-adjusted CPAP) has an additional therapeutic value in comparison with auto-CPAP alone. The combination therapy provided a better nocturnal oxygenation and a better control of sleep apnoea at altitude. Sleep efficiency and total sleep time also improved significantly, however without a difference in daytime performance [44].

As acetazolamide alone does not adequately control obstructive events as does CPAP alone not optimally control periodic breathing, the combined therapy can be of value in OSAS patients wanting to travel to altitude destinations [44].

The current literature thus suggests that CPAP treatment should preferably be continued in an auto-adjusting mode with acetazolamide as an add-on treatment to achieve better sleep efficiency and higher arterial oxygen saturation. CPAP devices are capable to adjust to the lower barometric pressure of altitude [45, 46]. If patients are unable to use a CPAP device, a personal fitted mandibular advancement device might be an alternative treatment option with or without combined acetazolamide therapy.

### Sleep disturbances in patients with chronic respiratory comorbidity

In comparison with healthy individuals, patients with COPD may be more prone to develop altitude related sleep and breathing disturbances, due to a pre-existing gas exchange impairment at sea level [12, 47].

The effect of moderate to high altitude on nocturnal oxygenation, breathing pattern and sleep quality was evaluated in 32 moderate to severe COPD patients without previous diagnosis of OSAS, living below 800 m. Patients were examined at a low altitude (490 m) before they spent 2 nights at an altitude of 1650 m and 2590 m in a randomised order. At each location, polysomnography was performed during the first night.

Results show a significant reduction in nocturnal oxygenation, decreasing from a median SpO_2_ of 92% at 490 m to 89% at 1650 m and 85% at 2590 m. Median AHI was 11.9/h at 490 m increasing to 23.3/h at 1650 m and 39.5/h at 2590 m, mainly due to central events. Conversely, obstructive events were more common at 490 m but did not change with increasing altitude. Sleep duration and sleep structure were impaired at 2590 m, with reduced sleep efficiency and longer wakefulness. Daytime vigilance evaluation at altitude showed a subtle reduction in alertness as compared with 490 m, however, no impairment of trail making performance, was observed. The Acute Mountain Sickness Score (Environmental Symptoms Questionnaire Cerebral score, AMSc) was slightly increased in the morning after the first night at 2590 m [47].

Tan et al. studied 32 moderate to severe COPD patients, during a 2 day and 2 night sojourn at 2048 m while receiving either nocturnal oxygen therapy (NOT) at 3L/min or sham therapy (room air) by nasal cannula. The objective of the study was to evaluate whether NOT prevents nocturnal hypoxaemia, reduces breathing disturbances and more importantly reduces the incidence of altitude related adverse health events. In comparison with sham therapy, NOT significantly improved nocturnal hypoxaemia (+ 9%), attenuated altitude induced sleep apnoea with a reduction of AHI by 29.7/h, and improved sleep structure and sleep efficiency with improved objective and subjective sleep quality at high altitude.

Most importantly, NOT reduced the incidence of AMS and intolerable dyspnoea, which were observed in eight patients (26%) on sham therapy versus one patient (4%) on NOT [12].

A previous study also reported about a quarter of moderate to severe COPD patients experiencing AMS or increased dyspnoea at 2590 m [17].

Altogether, these studies bring evidence of the benefit of nocturnal supplemental oxygen in patients with moderate to severe COPD travelling to moderate altitude, as it seems to limit symptoms of AMS as well as breathing disturbances, mainly due to central events. These studies also illustrate that a significant proportion of moderate to severe COPD patients are not fit to sojourn at high altitudes without appropriate precautions or preventive NOT, as about a quarter of them experienced AMS, relevant nocturnal hypoxemia or increased dyspnoea. Baseline nocturnal SpO_2_ was independently associated with nocturnal hypoxaemia and high altitude sleep disordered breathing, suggesting that a baseline nocturnal oximetry could be a minimal screening tool to identify the most susceptible COPD patients.

### Bullous or cystic lung disease, pneumatoceles and pneumothorax

Chronic bullae, chronic pneumatoceles or cysts are associated with emphysema, inflammatory or infectious process or genetic diseases. Concern arises that these bullae may expand and rupture with decreasing atmospheric pressure at altitude. Yet available data suggest that this concern is unwarranted [48]. Patients with bullous disease do not show and increased risk of pneumothorax. This may be explained by adequate communication channels between the bullae and the airways, enabling pressure equalisation [49].

This situation is however different for patients with an acute pneumothorax because air can become trapped in the pleural space through a valve effect and result in a tension pneumothorax. Therefore, patients with a recent history of pneumothorax should wait 2 weeks following radiographic resolution to travel to altitude [20].

Pulmonary lymphangioleiomyomatosis (LAM) is a rare disease affecting mostly women. LAM is characterised by cystic destruction of the lung parenchyma, resulting in dyspnoea, ventilatory and diffusion disorders and hypoxaemia. Patients with LAM have a high risk of spontaneous pneumothorax. The literature reports that 50–80% of LAM patients experience at least one pneumothorax with a recurrence rate of around 70%. Altitude travel is thus a genuine concern for these patients [50, 51]. Reduced barometric pressure may increase pulmonary cyst volumes up to 30%, possibly leading to cyst rupture and pneumothorax. Two previous retrospective surveys examined the risk of pneumothorax in LAM patients undertaking air travel. The estimated risk of pneumothorax was 1.1% and 2.2% respectively [51, 52]. In the study of Gonano et al., the incidence of pneumothorax increased by three with air travel in LAM patients. However, no studies were published on the impact of terrestrial altitude in patients with LAM [50].

### Pulmonary hypertension

Pulmonary hypertension (PH) may occur as a primary disorder or secondary to another underlying disease (COPD, obesity hypoventilation syndrome etc.). Precapillary PH is a condition haemodynamically defined as a mean PAP > 20 mmHg, along with a pulmonary arterial wedge pressure (PAWP) < 15 mmHg. In the absence of underlying relevant lung disease, the main forms are group I pulmonary arterial hypertension (PAH), with main causes being idiopathic, connective tissue disorders, chronic liver disease, human immune deficiency virus, drug or toxin induced, and group IV due to chronic thromboembolic pulmonary hypertension (CTEPH) [53].

Recent therapeutic advances in PH have led to improved quality of life and physical performance enabling these patients to travel more easily. To date, there are limited data about outcomes of PH patients when exposed to hypoxic environments during hours to days, as experienced by air travel or moderate to high altitude sojourns [54–56].

The first published case reports suggesting that PH might increase the risk of HAPE, described PH patients with anatomical anomalies or with chronic pulmonary hypertension due to permanent residency at high altitude (Monge’s disease) [57–60].

Beside HAPE susceptibility, an important concern is that patients with PH are at increased risk to develop acute RV dysfunction and cardiac ischemia. The sympathetic response induced by hypoxia leads to an elevated cardiac output and elevated blood pressure, as well as pulmonary arterial vasoconstriction, and this within minutes of exposure to altitudes from 1500 m.

These combined effects lead to increased PAP, increased RV afterload, and hence contribute to a deterioration of the RV function. RV dilatation may lead to decreased sub endocardial blood supply and ischemia. For patients with PH, a rapid, albeit small increase in PAP may impair the RV function without the ability to compensate the cardiac output, a concern that is particularly important during exertion. However, the severity of PH that predisposes to these problems is unknown.

In PH patients, belonging mostly to WHO functional class I to III, real air travel and simulated altitude studies suggest minimal clinical symptoms and minimal arterial desaturation at rest [61, 62]. The absolute level of hypoxaemia at rest may however not be the most appropriate measure to evaluate the cardiovascular risks of these patients, as it does not assess the potential stress of hypoxic environment on the RV function, neither does it take into account the supplemental stress when performing exercise.

As prospective data on exposure of patients with PH to real altitudes are lacking, the current recommendations for pulmonary hypertension state that patients with hypoxaemia at sea level, defined as PaO_2_ < 60 mmHg, or WHO functional class III to IV, should receive in flight oxygen administration. Similarly, such patients should avoid traveling to altitudes above > 1500–2000 m and, if unavoidable, they should use supplemental oxygen. However, these recommendations are not based on robust scientific evidence [53].

Seccombe et al. assessed the RV function at rest and during mild exercise at a simulated altitude of 2400 m, by echocardiography measurement. Fourteen clinically stable PAH group I (WHO class II to III) patients on specific therapy (Endothelin Receptor Antagonist or Phosphodiesterase 5-inhibitor) were exposed to normobaric hypoxia (FiO_2_ 15%) for 20 min and to room air. The study revealed that, despite a mean increase in pulmonary artery systolic pressure (PASP) during mild hypoxic exercise, no progressive RV deterioration neither at rest nor under mild exertion occurred. However, a lack of increase in the tricuspid annular plane systolic excursion (TAPSE) was observed during mild hypoxic exercise. This may indicate a reduced RV reserve and possibly reflect early signs of hypoxic decompensation.

The authors thus concluded that PAH patients with stable WHO functional class II and III, on specific treatment had no evidence of progressive RV deterioration during a short-term simulated altitude of 2400 m neither at rest nor during mild exercise. However, whether more prolonged periods of hypoxia can lead to deleterious effects, remains to be answered [63].

Exposure to normobaric hypoxia, with a simulated altitude of 2600 m, during right heart catheterisation did not significantly change invasive pulmonary haemodynamics at rest, in patients with group I (PAH) or group IV (CTEPH) despite a significant decrease in the arterial oxygen tension [PaO_2_] from 71 to 53 mmHg [64].

The effect of a real altitude 2-day sojourn (2048 m) on symptoms, exercise performance and cardiac function was evaluated in PH (functional class II to III) patients with and without supplemental oxygen. Altitude exposure induced significant hypoxaemia and reduced exercise performance without changes in pulmonary haemodynamics. Exercise performance was unaffected by supplemental oxygen [56].

Another randomised cross-over study examined the effect of normobaric hypoxia on exercise performance, clinical symptoms and pulmonary haemodynamics of 28 stable PH patients (group I and IV) with resting PaO_2_ ≥ 55 mmHg. Patients were randomly assigned to sham therapy (room air) or a hypoxic gas mixture (FiO_2_ 15%) while performing a cycle-ergometer constant work-rate exercise test (60% W max). A significantly reduced endurance was observed in hypoxaemic conditions compared to ambient air. However, similar dyspnoea was reported and similar pulmonary haemodynamics were observed [54].

Altogether these controlled studies included stable, relatively fit PH patients in WHO class II to III. Thus, these rather reassuring results may not be applicable for the less fit and more symptomatic patients.

Baseline disease severity assessment with pulmonary function tests (PFTs) and cardiac ultrasonography stays primordial when evaluating a PH patient wanting to undertake altitude travel. Because of limited evidence, the current PH guidelines stay the main reference [53]. HCT with or without mild exercise testing, could certainly be justified especially in patients with resting sea level SpO_2_ between 92 and 95%. When severe hypoxaemia at baseline, PaO_2_ < 60 mmHg, positive HCT or patients in WHO functional class III to IV, oxygen administration should be encouraged.

### Interstitial lung disease

In contrast with COPD, interstitial lung disease (ILD) at altitude has been less studied. Assuming similar effects of altitude on ILD and COPD patients is probably not correct, because the functional disorders in ILD and COPD patients differ greatly. Reduced carbon monoxide transfer capacity (TLCO), a hallmark of ILD and a marker of gas exchange dysfunction, has a much greater impact on oxygenation at altitude than FEV1.

One study compared the effect of simulated altitude (2438 m) by normobaric hypoxic testing in 15 ILD and 10 COPD patients at rest and during a limited 50-m walking test. In both groups, there was a statistically significant desaturation when breathing hypoxic air (FiO_2_ 15%) compared to ambient air. However, the ILD group showed a greater hypoxaemia than the COPD group**.** Even with an acceptable resting sea level oxygenation (PaO_2_ 83 mmHg), ILD patients showed a more profound desaturation, with a mean SpO_2_ of 87% (PaO_2_ 51 mmHg) at simulated altitude with worsening of these values at minimal exercise to a mean SpO_2_ of 79.5% (PaO_2_ 41 mmHg) [65].

Since the effect of hypoxaemia occurring during commercial air travel or altitude travel cannot accurately be predicted by resting blood gas analyses, ILD patients willing to travel to altitude would particularly benefit of formal hypoxic challenge testing. Because ILD patients are at risk of secondary PH as a complication of their underlying disease, their risk of RV dysfunction is also increased. Due to the lack of field studies of ILD at altitude, it is difficult to formulate strong recommendations for these patients. Just like the other respiratory diseases, baseline disease assessment by PFTs is important to assess baseline severity. Screening for underlying PH by cardiac ultrasonography could be justified, as well as HCT with or without mild exercise testing, in patients with resting sea level SpO_2_ between 92 and 95%.

### General assessment of patients with chronic respiratory conditions travelling to moderate and high terrestrial altitude

Clinical evaluation, with assessment of cardiac, muscular, neurological or other comorbidities, assessment of treatment adherence and baseline PFTs are key elements when giving advice to patients with underlying respiratory conditions planning to travel to moderate to high altitude.

Patients with asthma should be tested with spirometry while COPD, PH and ILD patients are to be additionally tested with assessment of lung volumes, lung diffusion capacity and in selected cases a blood gas analysis and exercise tests. This baseline functional assessment allows a first estimation of the risk associated with altitude exposure.

### Specific functional assessment

#### Hypoxic challenge test

The hypobaric hypoxic challenge test (HCT) is regarded as the reference for evaluating PaO_2_ at simulated altitude. However, because of the very limited availability of these laboratories, the test is not suitable for clinical practice [66].

The normobaric HCT, first described by Gong et al., has become the method of reference to assess the risk of hypoxaemia at altitude. During this test, the subject breathes a gas mixture (FiO_2_ of 15%, simulating an altitude of 2438 m) from a Douglas bag containing a decreased fraction of inspired oxygen or a gas mixing chamber for a duration of 20 min [67]. The clinician can adapt the desirable altitude by changing the fraction of inspired oxygen delivered to the subject so that the impact of higher altitudes can be evaluated as well. HCT testing is generally performed at rest, but this may not adequately simulate the physical activity during an altitude sojourn. Depending on the patients’ travel plans, a more precise hypoxemic risk assessment could be provided by including an exercise component during the HCT on a treadmill or a cycle-ergometer (e.g. 60% W max pred). Monitoring SpO_2_ during the HCT is mandatory. HCT should be proposed to patients with resting or exercise induced hypoxaemia or patients with severe COPD, ILD or PH, independently of the resting oxygenation level, based on clinical judgement. It is important to emphasize however, that simulated altitude exposure during this HCT test is of short duration (20 min) relative to what the subject will experience during their trip.

#### Prediction equations for altitude oxygenation

Prediction equations have been proposed as simpler alternatives to HCT to evaluate the risk of developing severe hypoxaemia [68]. The current British Thoracic Society guidelines provide four equations [20]. These equations have been derived from data obtained with COPD patients and use baseline PaO_2_ and baseline FEV1 (in litres or % predicted) [67–71]. Results of prediction equations show however poor accuracy with the actual individual hypoxic response (SpO_2_ and PaO_2_) during HCT and overestimate the need for supplemental oxygen [72]. Current literature suggests the use of these predictive equations solely as a screening tool.

Neither discouraging patients from travelling to altitude, nor prescribing unnecessary oxygen based on a predictive equation alone, can be recommended. Accurate oxygen prescription can only be done based on an individual assessment of the hypoxic response and cannot be derived from prediction equations. The HCT is the preferred method to identify patients who need supplemental oxygen and this test has a strong correlation with actual in flight data.

### Personalised treatment options for respiratory patients travelling to moderate and high altitude

Duration of planned travel, maximal planned altitude, altitude at which sleeping is possible and intensity of mandatory physical activity required by the stay has to be evaluated with the patient. Practical issues such as power supply and feasibility of oxygen supplementation, are also important considerations to take into account. This information should be integrated with the results of baseline PFTs and if indicated more specific tests in order to make a shared decision about the anticipated altitude sojourn. This way, complications can be reasonably prevented by optimisation of current medication and prophylactic treatment with acetazolamide or oxygen therapy.

Table 2 summarizes disease specific risk assessment and treatment options for patients with respiratory conditions travelling to terrestrial moderate and high altitude.

**Table 2:** Disease specific assessment and treatment options of respiratory patients travelling to terrestrial moderate and high altitude

Disease	Assessment	Treatment
COPD	Disease severity: spirometry and TLCO Exclude exacerbation Check treatment adherence 6MWD Consider HCT ± submaximal exercise test (if baseline SpO_2_ 92–95%) Consider screening for secondary PH by cardiac ultrasonography^a^	Advise patient about physical activity at moderate to high altitude: submaximal physical activity is generally well tolerated Take rescue medication Discuss feasibility of supplemental oxygen when positive HCT (PaO_2_< 50 mmHg) If co-existent PH: see recommendations below
Asthma	Disease severity: spirometry Exclude exacerbation Check treatment adherence Consider screening for EIB by bronchoprovocation test (high intensity exercise test or EVH test) Consider HCT ± submaximal exercise test (if baseline SpO_2_ 92–95%)	Provide written action plan in case of exacerbation Take rescue medication Avoid allergen exposure Advise patient to protect nose and mouth (scarf) to warm and humidify air Treat comorbid rhinitis, GER Consider pre-exercise/on demand short acting bronchodilator ± ICS
Sleep apnoea	Check CPAP device Check for underlying comorbid disease and consider more specific testing accordingly (e.g. COPD) Baseline nocturnal oximetry HCT ± submaximal exercise test (if baseline SpO_2_ 92–95%)	Sleep hygiene (sufficient and regular sleep) Continue CPAP device (auto-pilot set) Consider combination therapy with acetazolamide (2–3 × 250 mg/day) on top of CPAP device Acetazolamide alone (2–3 × 250 mg/day) better than no CPAP Consider mandibular advancement device if CPAP refused or unfeasible
Bullous/cystic lung disease	Consider HCT ± submaximal exercise test (if baseline SpO_2_ 92–95%)	Advise LAM patients about increased pneumothorax risk Discuss supplemental oxygen when positive HCT (PaO_2_ < 50 mmHg)
Pneumothorax	If recent history of pneumothorax, perform chest X ray	Postpone travel 2 weeks following radiographic resolution In case of recurrent pneumothorax, pleurodesis is recommended before travel
Pulmonary hypertension group I and IV	Disease severity: spirometry and TLCO 6MWDt Baseline cardiac ultrasonography Consider HCT ± mild submaximal exercise test (if baseline SpO_2_ 92–95%)	Discuss supplemental oxygen if: Severe hypoxaemia at sea-level (PaO_2_ < 60 mmHg) Positive HCT (PaO_2_ < 50 mmHg) WHO functional class III to IV
Interstitial lung disease	Disease severity: spirometry and TLCO 6MWD Consider HCT ± submaximal exercise test (if baseline SpO_2_ 92–95%) Consider screening for secondary PH by cardiac ultrasonography^a^	Discuss supplemental oxygen when positive HCT (PaO_2_ < 50 mmHg)

*COPD* chronic obstructive pulmonary disease, *TLCO* carbon monoxide transfer capacity, *HCT* hypoxic challenge test, *PH* pulmonary hypertension, *EIB* exercise induced bronchoconstriction, *EVH* eucapnic voluntary hyperpnoea, *ICS* inhaled corticosteroids, *GER* gastro-oesophageal reflux, *CPAP* continuous positive airway pressure, *LAM* lymphangioleiomyomatosis, *WHO* World Health Organization

^a^Decision for testing should be personalised depending on pre travel elements: duration of altitude exposure, maximal planned altitude, anticipated activity, electric supply, and feasibility of O_2_ supplementation

## Conclusions

This review article discusses six major respiratory disease groups and the possible risks faced by these respective patients when travelling to moderate to high altitude destinations. Strength of current recommendations is limited by the available evidence. Evidence derived from prospective controlled studies is limited by of the exclusion of patients with severe disease for the sake of safety. Thus, true risks of moderate to high altitude travel by patients with chronic respiratory conditions remains difficult to assess. Studies measuring the incidence of altitude related events in the most prevalent chronic respiratory conditions of varying severity are lacking. Epidemiological studies are needed to understand the incidence and risk factors of adverse events related to moderate altitude. Also, future clinical studies on the adaptation of patients with chronic respiratory conditions to moderate altitude should be designed to better delineate the effects of exercise compared to rest on physiologic and clinical outcomes.

Meanwhile, comprehensive personalised pre-travel assessment can ensure adequate measures to prevent monitor and mitigate worsening of underlying disease during travel.

## Data Availability

Not applicable.

## Abbreviations

AMS: Acute mountain sickness
HACE: High altitude cerebral edema
HAPE: High altitude pulmonary edema
COPD: Chronic obstructive pulmonary disease
PIO_2_: Partial pressure of inspired oxygen
PAO_2_: Partial pressure of alveolar oxygen
PaO_2_: Arterial oxygen pressure
HVR: Hypoxic ventilatory response
HPV: Hypoxic pulmonary arterial vasoconstriction
PAP: Pulmonary arterial pressure
GOLD: Global Initiative for Chronic Obstructive Lung Disease
FEV_1_: Forced expiratory volume in one second
6MWD: Six minute walking distance
LVEF: Left ventricle ejection fraction
RV: Right ventricle
FeNO: Fractional exhaled nitric oxide
BHR: Bronchial hyperresponsiveness
EIB: Exercise induced bronchoconstriction
FiO_2_: Fraction of inspired oxygen
PEF: Peak expiratory flow
EVH: Eucapnic voluntary hyperpnoea
OSAS: Obstructive sleep apnoea syndrome
CPAP: Continuous positive airway pressure
AHI: Apnoea-hypopnoea index
AMSc: Acute mountain sickness score
NOT: Nocturnal oxygen therapy
LAM: Lymphangioleiomyomatosis
PH: Pulmonary hypertension
PAP: Pulmonary arterial pressure
PAWP: Pulmonary arterial wedge pressure
CTEPH: Chronic thromboembolic pulmonary hypertension
WHO: World Health Organization
PASP: Pulmonary artery systolic pressure
TAPSE: Tricuspid annular plane systolic excursion
SDB: Sleep disordered breathing
PFT: Pulmonary function test
HCT: Hypoxic challenge test
ILD: Interstitial lung disease
TLCO: Carbon monoxide transfer capacity
